# Insights on the cGAS-STING Signaling Pathway During Herpesvirus Infections

**DOI:** 10.3389/fimmu.2022.931885

**Published:** 2022-07-01

**Authors:** Lishuang Deng, Zhiwen Xu, Fengqin Li, Jun Zhao, Zhijie Jian, Huidan Deng, Siyuan Lai, Xiangang Sun, Yi Geng, Ling Zhu

**Affiliations:** ^1^ College of Veterinary Medicine, Sichuan Agricultural University, Chengdu, China; ^2^ Key Laboratory of Animal Disease and Human Health of Sichuan Province, Sichuan Agricultural University, Chengdu, China; ^3^ College of Animal Science, Xichang University, Xichang, China

**Keywords:** cGAS-STING signaling pathway, herpesvirus, innate immune, antiviral response, viral evasion, immunotherapy

## Abstract

Herpesviruses belong to large double-stranded DNA viruses. They are under a wide range of hosts and establish lifelong infection, which creates a burden on human health and animal health. Innate immunity is the host’s innate defense ability. Activating the innate immune signaling pathway and producing type I interferon is the host’s first line of defense against infectious pathogens. Emerging evidence indicates that the cGAS-STING signaling pathway plays an important role in the innate immunity in response to herpesvirus infections. In parallel, because of the constant selective pressure imposed by host immunity, herpesvirus also evolves to target the cGAS-STING signaling pathway to inhibit or escape the innate immune responses. In the current review, we insight on the classical cGAS-STING signaling pathway. We describe the activation of cGAS-STING signaling pathway during herpesvirus infections and strategies of herpesvirus targeting this pathway to evade host antiviral response. Furthermore, we outline the immunotherapy boosting cGAS-STING signaling pathway.

## 1 Introduction

Herpesviruses belong to double-stranded DNA viruses with virions ranging in size from 120 to as much as 260 nm (1). They are ubiquitous worldwide and under a wide range of hosts, infecting almost all vertebrates, including humans, mammals, birds, and reptiles (2). The *Herpesviridae* family comprises three subfamilies: *alphaherpesvirinae*, *betaherpesvirinae*, and *gammaherpesvirinae*, composed of 115 viruses (3). Herpesviruses included in the *alphaherpesvirinae* subfamily have a wide range of hosts, a short reproduction cycle, rapid spread, and can destroy host cells. These viruses are reported to establish latent infections in sensory ganglia. Characteristics of the *betaherpesvirinae* subfamily members are markedly different from those of members of the *alphaherpesvirinae* subfamily. These viruses tend to latently infect lymphoreticular cells, secretory glands and kidneys. The hosts for members of the *gammaherpesvirus* subfamily often span different families. These viruses usually specifically infect T or B lymphocytes (4). Herpesviruses are ancient viruses that co-evolved with their hosts, and following exposure, they establish a lifelong infection that burdens human health and animal health (5). However, only fetuses and immunocompromised hosts usually exhibit severe clinical symptoms after herpesviruses infection (3). Notably, a few herpesviruses have certain interspecies transmission abilities: one kind of livestock and poultry may infect several types of virus, and one virus may also infect several animals, and even transmission infection between humans and animals occurs (6). The role of the innate immune system during herpesvirus infections is a worthy but grossly understudied aspect that calls for much more attention.

Innate immune system is the host’s first and most rapid line of defense in recognizing pathogens. It works by activating multiple intra- and extracellular signaling pathways to defend against pathogenic infection and protect organisms (7). Host recognition of pathogens relies on the pattern recognition receptors (PRRs) and their ligands, which include the conserved pathogen structures, pathogen-associated molecular patterns (PAMPs) and the newly reported host-derived damage-associated molecular patterns (DAMPs). Nucleic acids, lipoproteins, or polysaccharides associated with pathogenic structures are common PAMPs. In contrast, DAMPs are usually associated with cellular exposure to stress, and are therefore also known as alarmins (8, 9). Among infectious agents, viruses have a wide range of ecosystem impacts as an intracellular specialized pathogen and pose a serious threat to all living organisms (10, 11). Different PRRs are present in host cells to recognize viral nucleic acids (DNA or RNA). DNA is predominantly targeted by DNA-dependent activator of interferon response factors (DAI), cyclic GMP-AMP synthase (cGAS), absent in melanoma 2 (AIM2), interferon-gamma-inducible protein 16 (IFI16), interferon-inducible protein X (IFIX), and Toll-like receptors (TLRs) (7, 9, 12). Interestingly, TLR9 exclusively recognizes unmethylated DNA with CpG-motifs (CpG DNA) (9, 13). Viral RNA is mainly sensed by RIG-I-like receptors (RLRs) and some Toll-like receptors (TLR3/7/8) (14, 15).

cGAS plays a prominent role in viral DNA sensing and is rapidly activated by cytosolic DNA. Subsequently, the activated cGAS promotes the synthesis of 2’, 3’-cGAMP. Then 2’, 3’-cGAMP activates a protein located on the endoplasmic reticulum (ER), stimulator of interferon genes (STING) (16, 17). Subsequently, STING mediates several downstream signaling cascades to protect the host from pathogen invasion (18). Numerous studies have suggested that the cGAS-STING signaling pathway, mainly responsible for cytoplasmic DNA sensing, plays an important role in host innate immunity against viruses. In addition to recognizing and sensing DNA virus infection, this pathway has been reported to be involved in restricting the infection of RNA viruses (9, 19).. At the same time, because of the constant selective pressure imposed by host immunity, viruses are also evolving to target the cGAS-STING signaling pathway to inhibit or evade the host immune response (7, 11). Numerous recent studies have shown that the cGAS-STING signaling pathway is an ideal target for therapeutic intervention in diseases, especially in cancer and tumors (20–23), which may provide new ideas for combating herpesvirus.

In the current review, we insight on the classical cGAS-STING signaling pathway. Then we describe the activation of cGAS-STING signaling pathway during herpesvirus infections and strategies of herpesvirus targeting this pathway to inhibit and evade the host antiviral response. Finally, we outline the immunotherapy enhancing cGAS-STING signaling pathway.

## 2 Signaling in the Classical cGAS-STING Signaling Pathway

**Figure 1:**
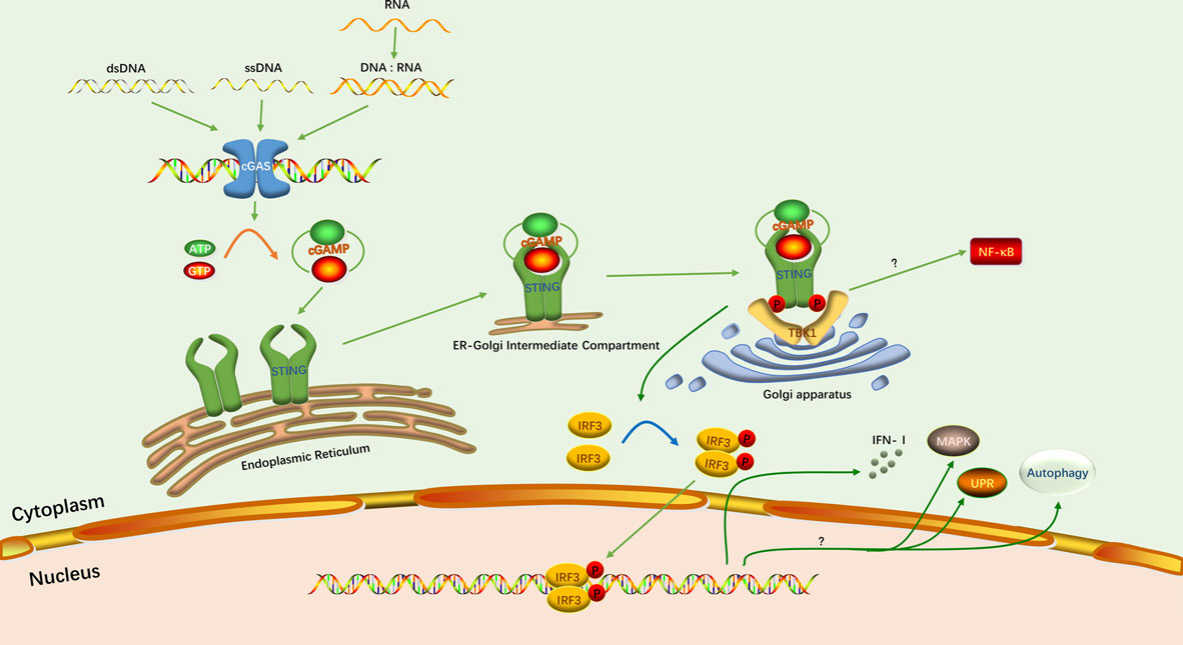
The classical cGAS-STING signaling pathway. The green arrows indicate the activation of this pathway and induction of the IFN-I response. The yellow arrow indicates the synthesis of cGAMP. The blue arrow indicates the phosphorylation of IRF3. Protein phosphorylation is depicted as P. The question mark indicates that the specific mechanism has not been elucidated.

cGAS-STING signaling pathway is one of the important pattern recognition and effector pathways in host innate immunity. The signaling of classical cGAS-STING signaling pathway is generally thought to consist of three main phases: nucleic acid-sensing, intracellular signal transduction, and immune response activation (**Figure 1**).

### 2.1 Nucleic Acid Sensing

Viral DNA is sensed by specific sensor proteins which mainly present in the cytoplasm of host cells. Sensor proteins recognize viral DNA and activate an innate immune response against the virus (9). cGAS is one of the most important DNA sensors, and in particular its ability to induce type I interferon (IFN-I) responses has been well documented (24, 25). The DNA-sensing and enzymatic activity of cGAS were first described in 2013 (25, 26). cGAS belongs to the nucleotidyltransferase family, and uses purines or pyrimidines to synthesize linear or cyclic dinucleotide or trinucleotides to activate downstream signaling molecules (27). cGAS is activated by double-stranded DNA (dsDNA) and recognizes dsDNA in a length-dependent manner. cGAS binding to short dsDNA less than 20 bp results in incomplete formation of cGAS dimers, which prevent the formation of stable complexes. On the contrary, binding to large fragments of dsDNA induces high affinity of cGAS for the target nucleic acid and the formation of a network of cGAS-DNA oligomers (24, 28, 29). cGAS binds directly to dsDNA through its zinc-ribbon domain. Subsequently, its conformation changes and induces its enzymatic activity. Then, cGAS utilizes its enzymatic function to catalyze adenosine triphosphate (ATP) and guanosine triphosphate (GTP) into a second cyclic messenger, 2’, 3’- cGAMP (11, 30). cGAS has been reported to bind to single-stranded DNA (ssDNA), but only induces small amounts of cGAMP (31). Hybrid DNA and RNA, as well as stem-like ssDNA, can also activate cGAS, but with lower potency (9). In addition, cGAS can bind to dsRNA, but this does not induce cGAMP production (24).

### 2.2 Intracellular Signal Transduction

STING is an important junctional protein in the cGAS-STING signaling pathway and is the downstream ligand of cGAMP. STING utilizes its amino-terminal transmembrane domain to anchor it to the ER membrane, with the carboxyl terminus projecting into the cytoplasm (32). With or without ligand, STING exists as a dimer on the ER membrane (33–36). When cGAMP is synthesized, the STING dimer binds to a molecule of cGAMP through hydrophobic interactions and hydrogen bonding (33, 34). Then STING undergoes conformational change, activation and release of the carboxy-terminal tail (CTT) domain, which resembles a TANK binding kinase 1 (TBK1) substrate and permits recruitment of the downstream TBK1 (37). The complex of cGAMP and STING will be transferred from the ER to the Golgi apparatus by the ER-Golgi intermediate compartment (ERGIC) to further recruit TBK1 (38). Subsequently, the CTT domain exposed by STING is inserted into the groove formed between the kinase domain and scaffold dimerization domain (SDD) of TBK1 dimer, which is tightly bound (39, 40). TBK1 in turn promotes the phosphorylation of the CTT domain, which causes the recruitment of the interferon regulatory factor 3 (IRF3) (41).

### 2.3 Immune Response Activation

The recruited IRF3 forms a dimer after phosphorylation by TBK1. It then enters the nucleus to induce transcription of IFN-I (42). Triggering the IFN response is the most important function of the cGAS-STING signaling pathway. However, studies have confirmed that this pathway has functions not related to IFN (10). In inflammation, the cGAS-STING signaling pathway is associated with activation of the nuclear factor-kB (NF-κB) pathway. Although the exact mechanism is currently unknown, STING and TBK1 are indispensable for inducing NF-κB signaling pathway (43, 44). In addition, there are three other downstream responses of cGAS-STING signaling pathway have been reported: mitogen-activated protein kinase (MAPK) pathway, autophagy, and the unfolded protein response (UPR) (45–47).

## 3 The cGAS-STING Signaling Pathway in Herpesvirus Infections

**Figure 2:**
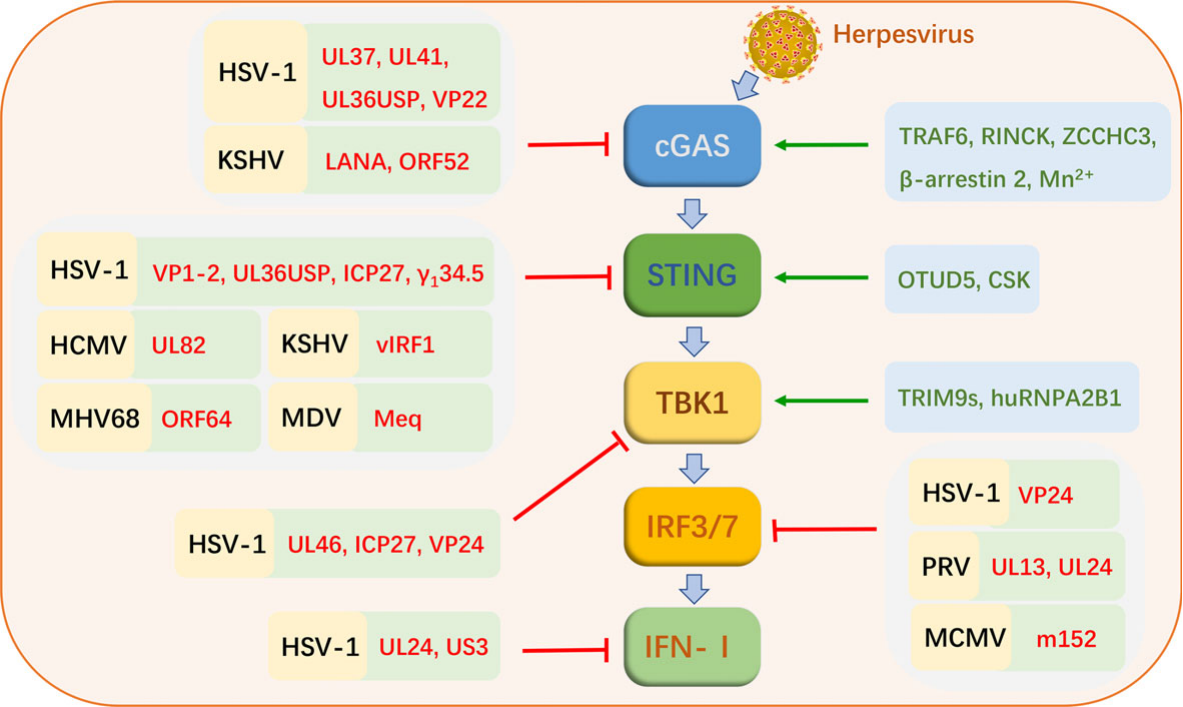
Strategies of cGAS-STING signaling pathway activation and virus evasion during herpesvirus infections. Black letters represent herpesviruses. Red letters indicate the proteins of these viruses. Green letters indicate cellular proteins that favor cGAS-STING signaling pathway activation.

The cGAS-STING signaling pathway is activated during herpesvirus infections (**Figure 2**). As herpesviruses co-evolved with hosts, some members of viruses have acquired their unique mechanisms and strategies to evade innate antiviral immunity, which mainly interferes with different factors in the cGAS-STING signaling pathway (**Table 1** and **Figure 2**).

**Table 1:** Herpesvirus evasion of innate immunity *via* cGAS-STING signaling pathway.

Subfamily	Virus	Proteins	Target	Mechanism	Reference
*Alphaherpesvirinae*	HSV-1	UL46	TBK1	Targeting TBK1 and inhibiting TBK1 dimer formation to suppress IRF3 activation.	(48)
		UL41	cGAS	Degrading cGAS *via* its RNase activity.	(49)
		UL37	cGAS	Deamidating cGAS to catalyze cGAMP synthesis.	(50)
		UL24	IFN-β promoter	Inhibiting the activation of the promoters of IFN-β and IL-6.	(51)
		VP24	TBK1 and IRF3	Disrupting the interaction between TBK1 and IRF3 to impaire IRF3 activation.	(52)
		VP22	cGAS	Interacting with cGAS and inhibiting the enzymatic activity of cGAS.	(53)
		VP1-2	STING	Directly interacting with STING and promoting the deubiquitination of STING to evade antiviral responses.	(54)
		UL36USP	cGAS and STING	Blocking the activation of the IFN-β promoter to evade host antiviral innate immunity by targeting cGAS and STING.	(55)
		ICP27	TBK1 and STING	Interacting with TBK1 and STING *via* its RGG motif.	(56)
		γ_1_34.5	STING	Directly interacting with STING to further inhibit the activation of TBK1.	(57)
		US3	β-catenin	Hyperphosphorylating β-catenin *via* its kinase activity.	(58)
	PRV	UL13	IRF3	Phosphorylating IRF3 and disrupting IRF3 binding to downstream factor promoter.	(59)
		UL24	IRF7	Inhibiting the production of IFN-β by targeting IRF7.	(60)
*Betaherpesvirinae*	HCMV	UL82	STING	Interacting with STING and iRhom2, and disrupting the binding of STING to TRAPb to prevent the transfer of STING to the Golgi apparatus. Additionaly inhibiting the recruitment of downstream signaling molecules.	(61)
	MCMV	m152	IRF3	Inhibiting STING-mediated IRF signaling.	(62)
*Gammaherpesvirinae*	KSHV	vIRF1	STING	Blocking the interaction of STING with TBK1, and then inhibiting the phosphorylation and activation of STING to suppress cGAS-STING-mediated antiviral immunity.	(63)
		LANA	cGAS	Directly binding to cGAS and inhibits the downstream phosphorylation of TBK1 and IRF3.	(62)
		ORF52	cGAS	Directly inhibiting cGAS enzymatic activity.	(64)
	MHV68	ORF64	STING	ORF64 DUB active site mutant is associated with impaired delivery of viral DNA to the nucleus.	(65)
	MDV	Meq	STING	Blocked the recruitment of downstream TBK1 and IRF7 to the STING.	(66)

### 3.1 Activation of the cGAS-STING Signaling Pathway During Herpesvirus Infections

The cGAS-STING signaling pathway is critical to drive the initial IFN-I response and limite herpesvirus infection. Multiple herpesviruses have been shown to trigger this pathway, including herpes simplex virus-1 (HSV-1), oncolytic herpes simplex viruses (oHSV), pseudorabies virus (PRV), human cytomegalovirus (HCMV), mouse cytomegalovirus (MCMV), Kaposi’s sarcoma-associated herpesvirus (KSHV) (63, 67–72). HSV-1 is the most widely reported member associated with cGAS-STING signaling pathway in various studies.

In addition to being directly activated by viral DNA, the cGAS-STING signaling pathway can undergo different modifications for optimal signaling of HSV-1 infection, including ubiquitination and phosphorylation. E3 ubiquitin ligases, TRAF6 and RINCK (also known as TRIM41), promotes ubiquitination and activation of cGAS in IFN-I production during HSV-1 infection. IFN-β production is inhibited in TRAF6-knockdown cells infected with HSV-1. IFN-I production also is inhibited the RINCK−deletion cells upon HSV-1 infection, which is associated with the phosphorylation of TBK1 and IRF3 (73, 74). TRIM9s, believed to function as E3 ubiquitin ligases, are also identified as positive IFN-I regulators. Upon cellular sensing of HSV-1 DNA, TRIM9s undergo ubiquitination, and promote the recruitment and activation of TBK1, leading to IRF3 activation and IFN-I production (75). Deubiquitinases (DUB) also play an important role in the cGAS-STING signaling pathway. The ovarian tumor deubiquitinase 5 (OTUD5) can target STING to cleave its polyubiquitin chain and promote its stability. In cells with knockdown of OTUD5, degradation of STING in the HSV-1 triggered cGAS-STING signaling pathway is accelerated, thereby inhibiting IFN-I production (76).

Other cellular proteins also contribute to activating the cGAS-STING signaling pathway during HSV-1 infection. Heterogeneous nuclear ribonucleoprotein A2B1 (hnRNPA2B1) is a newly identified intranuclear DNA sensing protein. hnRNPA2B1 binds intranuclear viral DNA and transfers it to the cytoplasm during HSV-1 infection. It then activates TBK1 to promote the expression of downstream antiviral genes (77). ZCCHC3, a CCHC-type zinc-finger protein, positively regulates cGAS. Upon HSV-1 infection, binding of ZCCHC3 to viral DNA promotes cGAS binding to DNA, thereby facilitating cGAS activation. Conversely, ZCCHC3 deficiency leads to suppression of downstream antiviral gene expression triggered by viral DNA, which makes it easier for the virus to proliferate in the host (78). The nonreceptor tyrosine kinase CSK is also important for sensing of DNA viruses in the cGAS-STING signaling pathways. Deletion of CSK inhibits the expression of multiple downstream antiviral genes in the cGAS-STING pathway, including *ifnb1*, *ifvn4*, and *isg56*. Following HSV-1 infection, CSK phosphorylates STING at Y240 and Y245 after the phosphorylation of STING at S366 by TBK1 (79). β-arrestin 2, a multifunctional adaptor, promotes the clearance of HSV-1 and vesicular stomatitis virus (VSV) and virus-induced production of IFN-β in macrophages. β-arrestin 2 targets cGAS and promotes dsDNA-cGAS interactions as well as cGAMP production to induce STING expression and innate immune responses (80). A recent study reported that Mn^2+^ is a positive regulator of cGAS. Mn^2+^ promotes dsDNA binding to cGAS and increases the enzymatic activity of cGAS, which is beneficial to cGAS sensing low concentration of dsDNA. Furthermore, Mn^2+^ also promotes cGAMP binding to STING and increases the activity of STING. In the Mn-deficient mouse model, the production of antiviral factors is deprived, which is more conducive to viral infection and replication (81).

### 3.2 Evasion of the cGAS-STING Signaling Pathway by Herpesvirus

Herpesviruses are remarkable pathogens, which encode at least 70 functional proteins. The member known to encode the largest number proteins is HCMV that encode about 170 proteins. They can efficiently utilize their proteins and host components to evade host immunity and achieve proliferation and egress. With the in-depth study of herpesvirus-host interactions, we have understood how herpesviruses evade the cGAS-STING signaling pathway (**Table 1** and **Figure 2**).

#### 3.2.1 Alphaherpesviruses Evade the cGAS-STING Signaling Pathway

HSV-1 is the first virus reported triggering the cGAS-STING signaling pathway, and it encodes many proteins targeting this pathway to inhibit the host antiviral responses (82–84). HSV-1 tegument protein UL46 targets TBK1, and inhibits TBK1 dimer formation and its interaction with IRF3. Ultimately, it inhibits the activation of IRF3 and the production of IFN-I. UL46-deficient HSV-1 enhances the production of IFN-I and inhibits viral replication. Not surprisingly, the proliferation of UL46-deficient HSV-1 is stronger in TBK1-deficient cells (48). It has been proven that HSV-1-encoded UL41 inhibits host perception of the virus by targeting cGAS. The detailed mechanism is that UL41 has RNase activity to degrade cGAS. Deletion of UL41 promotes activation of the IFN-β promoter and production of IFN-β (49). UL37, another tegument protein of HSV-1, also targets cGAS to escape the host antiviral response. UL37 has been shown to have a deamidating effect, which disrupts the catalytic function of cGAS by deamidating it to inhibit the synthesis of cGAMP. Deamidase-deficient HSV-1 triggers a strong immune response in mice, leading to inhibition of viral replication (50). UL24 of HSV-1, an essential protein that promotes herpesvirus replication. It was demonstrated that UL24 inhibits the activation of the promoters of IFN-β and interleukin-6 (IL-6), thus facilitating HSV-1 evasion of antiviral responses and achieving efficient viral proliferation (51). The VP24 of HSV-1 is a newly identified protein that inhibits the production of IFN-β. VP24 inhibits the expression of DNA-induced IFN-β and the activation of the IFN-β promoter in the cGAS-STING signaling pathway. The specific mechanism is that VP24 impaires IRF3 activation and IFN-I production by disrupting the interactions between TBK1 and IRF3 (52). HSV-1 tegument protein VP22 acts as an antagonist of the IFN-I pathway. VP22 interacts with cGAS and inhibits the enzymatic activity of cGAS from evading innate immune responses persistently (53). VP1-2 of HSV-1 is a protein with DUB activity that interacts directly with STING. VP1-2 promotes the deubiquitination of STING to evade antiviral responses, especially in the brain. VP1-2 DUB activity deficient HSV-1 promotes IFN-stimulated gene expression and IFN production, and inhibits the replication of this virus. This is due to promoted ubiquitination of STING and phosphorylation of TBK1 as well as IRF3 (54). Another protein with DUB activity, HSV-1 ubiquitin-specific protease (UL36USP), is thought to primarily inhibits the activation of the IFN-β promoter to evade host antiviral responses by targeting cGAS and STING (55). HSV-1 inhibits IFN-I induction in human macrophages *via* the conserved herpesvirus protein ICP27. ICP27 targets TBK1 and STING in the cGAS-SING signaling pathway. The activity of TBK1 is important for this process (56). γ_1_34.5 of HSV-1 directly interacts with STING to inhibit the activation of TBK1. During viral infection, the γ_1_34.5 protein disrupts the transport of STING from the ER to the Golgi apparatus, which leads to downregulation of IRF3 and IFN responses (57). β-catenin is a crucial protein that promotes the expression of IFN-I. US3 encoded by HSV-1 inhibits IFN-I production by hyperphosphorylating β-catenin *via* its kinase activity (58).

In animal herpesviruses, research into the mechanisms of evasion of the cGAS-STING signaling pathway is just beginning. There are limited reports. PRV is a DNA virus that seriously affects the health of the pig industry worldwide. The proteins of PRV inhibit the production of IFN-I by targeting IRFs in the cGAS-STING pathway. PRV UL13 potently inhibits IFN-β production by phosphorylating IRF3 and disrupting IRF3 binding to downstream factor promoter (59). Another study showed that UL24 of PRV inhibits the production of IFN-β by targeting IRF7. UL24-deleted PRV strain significantly increased the transcription levels of IFN-I in PK-15 cells (60).

#### 3.2.2 Betaherpesviruses Evade the cGAS-STING Signaling Pathway

Not only alphaherpesviruses can use their proteins to escape host innate immunity through the cGAS-STING signaling pathway, but some members of *the betaherpesvirinae* subfamily have also evolved their escape mechanisms. Tegument protein UL82 plays an important role in immune evasion of HCMV, which directly targets STING for inhibition of innate antiviral responses. UL82 facilitates the replication of HCMV. Conversely, inhibition of UL82 expression or deletion of UL82 results in high levels of intracellular antiviral genes. UL82 inhibits the STING signaling pathway by interacting with STING and iRhom2 and by disrupting the binding of STING to TRAPb to prevent the transfer of STING to the Golgi apparatus. In addition, UL82 inhibits the recruitment of downstream signaling molecules (TBK1 and IRF3) (61). MCMV m152 protein targets the IFN-I response by inhibiting STING-mediated IRF signaling (62).

#### 3.2.3 Gammaherpesviruses Evade the cGAS-STING Signaling Pathway

The proteins of several gammaherpesviruses have been reported to inhibit the cGAS-STING signaling pathway. The viral IFN regulatory factor 1 (vIRF1) of KSHV was shown to be an important negative regulator of STING. vIRF1 blocks the interactions of STING with TBK1 and then inhibits the phosphorylation and activation of STING to suppress cGAS-STING-mediated antiviral immunity (63). The latency-associated nuclear antigen (LANA) encoded by KSHV is also one of the proteins that facilitate viral escape. LANA inhibits the clearance of KSHV from host cells by directly binding to cGAS to inhibit the phosphorylation of TBK1 and IRF3 (62). KSHV tegument protein, ORF52, also directly targets cGAS to inhibit its enzymatic activity and inhibit the sensing of viral DNA (64). In the murine gammaherpesvirus 68 (MHV68) infection model, deletion of ORF64 DUB has been shown to inhibit viral replication and proliferation. And mutation of the ORF64 DUB active site also diminishes the ability of the virus to establish latent infection in mice. However, this phenomenon does not occur in mice lacking STING, which indicates that MHV68 mainly escapes the host immune responses by inhibiting the function of STING through the ORF64 DUB (65). Meq, the major oncoprotein encoded by Malik’s disease virus (MDV), is also a negative regulatory protein of STING. Overexpression of Meq significantly inhibits antiviral responses. In contrast, knockdown of Meq significantly increased the expression levels of antiviral genes and IFN-β in cells infected with MDV. The specific mechanism is that Meq blocks the recruitment of downstream TBK1 and IRF7 to the STING complex to inhibit IFN-β production (66).

## 4 Immunotherapy Boosting cGAS-STING Signaling Pathway

The cGAS-STING signaling pathway is indispensable for the sensing of herpesvirus in host innate immunity, which makes this pathway an important candidate target for the therapy of herpesvirus infections. In fact, numerous studies have explored the immunotherapy targeting cGAS-STING signaling pathway, especially in tumor and cancer treatment, showing great therapeutic potential. Immunotherapy boosting the cGAS-STING pathway mainly includes STING agonist, improved delivery system and combination therapy. We outline these therapeutic approaches below, which may provide new ideas for against herpesvirus infections.

### 4.1 STING Agonist

#### 4.1.1 Cyclic Dinucleotide

Cyclic dinucleotides (CDNs) have been verified as a class of cGAS-STING signaling pathways agonist involved in the immune system in prokaryotic cells and mammalian cells. CDNs consist of cyclic di-GMP (cdGMP), cyclic di-AMP (cdAMP), cyclic AMP-GMP (cGAMP) (20). cGAMP is one kind of the most classical CDNs, including 2’, 3’-cGAMP, 2’, 5’-cGAMP, 3’, 3’-cGAMP and 3’, 5’-cGAMP. CDNs have various applications in antitumor immunity (20–23). For instance, intra-tumoral injection of 2’, 3’-cGAMP significantly retards tumor growth in B16F10 mice (85).

Except for natural CDNs, synthetic CDNs with better properties are developed in recent years, such as ADU-S100 and ADU-V19. ADU-S100 (ML RR-S2 CDN), also known as MIW815, is a dithio CDN with high affinity to human STING. Therefore, it becomes the first STING agonist to enter clinical trials for advanced metastatic solid tumors or lymphomas. ADU-V19 (RR-S2 cGAMP) is similar to ADU-S100 and targets human STING (21).

#### 4.1.2 Non-CDN Agonist

Recent studies indicate that non-CDN agonists also play an important pharmacological role in immunotherapy targeting the cGAS-STING signaling pathway. 5,6-dimethylxanthenone-4-acetic acid, also named DMXAA or ASA404, is a flavonoid. DMXAA is a STING non-CDN agonist and is found to be selective for STING. In T cells of B6 mice, the IFN-I response is considerably enhanced after treatment with DMXAA (86). However, DMXAA is not effective in phase III trials in patients with non-small cell lung cancer (87). Together, DMXAA induces mice STING rather than human STING to enhance the innate immune response, which may be caused by the structural differences between the two STINGs. Amidobenzimidazole (ABZI) is another STING non-CDN agonist. After dimerization of ABZI (di-ABZI) with a 4-carbon butane linker, the binding affinity of di-ABZI to STING is significantly enhanced (22).

### 4.2 Improved Delivery System

cGAS-STING agonists are usually soluble, susceptible to enzymatic hydrolysis, and negatively charged, limiting their therapeutic efficacy (21). Therefore, efficient biomaterial delivery systems are essential to optimize the therapeutic strategy and enhance the biotherapeutic efficacy of these agonists. There are a variety of cGAS-STING agonist delivery systems, three of which have been extensively studied: nanocarrier, microparticle, and hydrogel.

Liposome is the first FDA-approved nano-biotherapeutic carrier. Liposome nano-delivery systems containing STING agonists can target specific cells for cell activation. Liposome nanoparticles encapsulating cdGMP target to draining lymph nodes. The therapeutic efficacy of the nano-delivery system is considerably improved in murine compared with cdGMP alone (88). Polymer nanoparticle is another promising nano-delivery system. Studies have demonstrated that synthetic polymeric nanocarrier, PC7A NP, induces robust immune response through the cGAS-STING pathway in mice (89).

Microparticle, a recently developed STING agonist delivery system, include tumor cell-derived microparticle (TMP) and acid-sensitive acetylated dextran (Ace-DEX) polymer microparticle (Ace-DEX MP) (21). TMPs are microparticles produced by apoptotic cancer cells that promote the production of IFN-I by activating the cGAS-STING signaling pathway. Ace-DEX MP is another MP delivery system and is a powerful subunit vaccine delivery system. Ace-DEX MPs encapsulating 3’, 3’-cGAMP in combination with a soluble TLR7/8 agonist elicit robust cytokine responses in mouse bone marrow-derived dendritic cells (90). Another study showed that in influenza vaccine, Ace-DEX MPs containing 3’, 3’-cGAMP induce the strongest antibody production and immune responses *in vivo* (91).

Hydrogel is another common delivery system that forms viscoelastic gels by binding and retaining water. Cross-linked hyaluronic acid (HA) hydrogel loaded with 2’, 3’-cdAM (PS)2 (Rp,Rp) significantly enhances the therapeutic effect of tumors (92). cGAMP-loaded linear HA hydrogel promotes the IFN-I production in macrophages (21, 93). Matrigel is a thermally responsive hydrogel. Matrigel combined with a STING agonist is beneficial for the treatment of localized tumors in mice with head and neck squamous cell carcinoma (94). STINGel is a peptide hydrogel for intra-tumoral CDN delivery. In mice with oral cancer, CDN-loaded STINGel activates the cGAS-STING pathway to promote immune response (95).

### 4.3 Combination Therapy

STING agonists and optimized delivery systems target and activate the cGAS-STING signaling pathway to promote immunotherapy of tumors and other diseases. However, a growing body of research has shown that combination immunotherapy targeting the cGAS-STING signaling pathway maximizes the efficacy of STING agonists in immunotherapy of disease and reduce drug negative impacts.

#### 4.3.1 STING Agonist Vaccine Adjuvant

Appropriate adjuvants play a major role in enhancing the specific immunity of vaccines. To increase the effectiveness of vaccines, various adjuvants have been developed to promote innate immunity. Among them, STING agonists have shown potential as adjuvants in vaccinology. STINGVAX (ML-RR-S2-CDA) is believed to be the first cancer vaccine to utilize a STING agonist as an adjuvant, which comprises cancer cells secreting granulocyte-macrophage colony-stimulating factor and ADU-S100 (22). STINGVAX shows strong antitumor effects in mouse models (20, 22, 23). Another study showed that using 2’, 3’-cGAMP as an adjuvant of the recombinant hemagglutinin (rHA) influenza vaccine increases rHA-specific antibody titers in adult mice (96). Furthermore, influenza vaccine using 3’, 3’-cGAMP as an adjuvant elicits potent immune response with protection against lethal influenza challenge (91).

#### 4.3.2 Combination With Chemotherapy

Synergistic administration reduces the required dose of each drug and narrows the side effects of drugs. STING agonists combined with other chemotherapy drugs have achieved good efficacy in anti-tumor. DMXAA synergistically promotes cell death in mouse sarcomas when treated with cyclophosphamide (20). The combination of the chemotherapy drug fluorouracil (5FU) and cGAMP was beneficial in reducing colon cancer tumor activity (97). Importantly, side effects of both cyclophosphamide and 5FU are reduced. In addition, paclitaxel and carboplatin can be combined with STING agonists to produce better therapeutic effects (98, 99).

#### 4.3.3 Combination With Immune Checkpoint Blockade and Other Therapies

Usually, immune checkpoint blockers (ICBs) target cytotoxic T lymphocyte-associated protein 4 (CTLA4) and programmed cell death 1 (PD-1) or its ligand PD-L1 (23). Combination therapy of STING agonists and ICBs has attracted considerable attention in recent years. cGAMP combined with anti-CTLA4 and anti-PD-1 mAb treatment inhibits tumor growth (100). Moreover, cdGMP combined with anti-CTLA-4/PD-1, and dithio-cdGMP combined with anti-PD-L1 have been verified to enhance the anti-tumor effect of ICB therapy (20). STING agonists are also combined with radiotherapy, chimeric antigen receptor T cell therapy and surgery to achieve better therapy outcomes (22, 23). Indeed, the combination of STING agonists with multiple immunotherapies tends to induce the strongest immune responses, which may be one of the main strategies for treating diseases in the future.

## 5 Conclusions and Future Perspectives

In the current review, we describe the cGAS-STING signaling pathway in herpesvirus infections and outline the immunotherapy boosting this pathway. The cGAS-STING signaling pathway is essential for immunological defense of the host against herpesvirus infections. In parallel, herpesviruses also evolves to target the cGAS-STING signaling pathway to inhibit or escape the host innate immune response, thereby promoting their replication. Given the importance of the cGAS-STING signaling pathway in the sensing of herpesvirus in innate immunity, it is an attractive antiviral strategy to develop and utilize immunotherapy that enhances the cGAS-STING signaling pathway. In addition, since our review is limited to the classical cGAS-STING signaling pathway in herpesvirus infections, exploring the potential crosstalk between this pathway and other pathways of the innate immune system during viral or other pathogenic infections may be a matter of great interest in the future, such as MAPK pathway and autophagy.

## Author Contributions

LZ, LD, and ZX provided ideas. LD wrote the manuscript and designed the figures. ZX, FL, JZ, ZJ, HD, SL, XS, YG, and LZ reviewed and modified the manuscript. All authors contributed to the article and approved the submitted version.

## Funding

This article was supported by the Sichuan Province’s “14th Five-Year Plan” Sichuan Pig Major Science and Technology Project (No. 2021ZDZX0010) and the Key R&D Program in Rural Areas of Sichuan Provincial Department of Science and Technology (No. 2020YFN0147).

## Conflict of Interest

The authors declare that the research was conducted in the absence of any commercial or financial relationships that could be construed as a potential conflict of interest.

## Publisher’s Note

All claims expressed in this article are solely those of the authors and do not necessarily represent those of their affiliated organizations, or those of the publisher, the editors and the reviewers. Any product that may be evaluated in this article, or claim that may be made by its manufacturer, is not guaranteed or endorsed by the publisher.
